# How will e-cigarettes affect health inequalities? Applying Bourdieu to smoking and cessation

**DOI:** 10.1016/j.drugpo.2018.01.009

**Published:** 2018-04

**Authors:** Frances Thirlway

**Affiliations:** Durham University, Anthropology Department, The Palatine Centre, Stockton Road, Durham DH1 3LE, UK

**Keywords:** Smoking cessation, E-cigarettes, Bourdieu, Distinction, Class, Habitus, Stigma

## Abstract

This paper uses the work of Bourdieu to theorise smoking and cessation through a class lens, showing that the struggle for distinction created the social gradient in smoking, with smoking stigma operating as a proxy for class stigma. This led to increased policy focus on the health of bystanders and children and later also to concerns about electronic cigarettes. Bourdieu’s concept of habitus is deployed to argue that the e-cigarette helps middle-class smokers resolve smoking as a symptom of cleft habitus associated with social mobility or particular subcultures. E-cigarette use is also compatible with family responsibility and sociable hedonism; aspects of working-class habitus which map to the ‘practical family quitter’ and the ‘recreational user’ respectively. The effectiveness of class stigma in changing health behaviours is contested, as is the usefulness of youth as a category of analysis and hence the relevance of concerns about young people’s e-cigarette use outside a class framework of smoking and cessation. With regard to health inequalities, whilst middle-class smokers have in class disgust a stronger incentive to quit than working-class smokers, there is potential for tobacco control to tap into a working-class ethos of family care and responsibility.

## Introduction

The advent of electronic (‘e’-) cigarettes has disrupted existing narratives (Stimson, Thom, & Costall, 2014) and there is continuing controversy as to whether they are helpful to tobacco control (McNeill et al., 2015; Nutt et al., 2016). In this article I argue that the impact of e-cigarettes on smoking prevalence and cessation rates in high-income countries can best be theorised through a class lens. Drawing on a range of disciplines including the social sciences, history, politics and public health, I show that Bourdieu’s ‘struggle for distinction’ has driven the social gradient in smoking in high-income countries. I then explore how different aspects of class habitus are more or less compatible with smoking, cessation and e-cigarette use as classed cultural practices and identify different categories of e-cigarette use. Bourdieu suggested that ‘the logic of research is inseparably empirical and theoretical’ and I ground my theoretical analysis in fieldwork on smoking and the determinants of cessation undertaken in working-class areas of the North of England since 2012, in accordance with his argument that ‘one cannot think well except through theoretically constructed empirical cases’ (Bourdieu & Wacquant, 1992, p. 159–60),

### Bourdieu and health

French sociologist Pierre Bourdieu’s work was essentially concerned with class; he argued that a system of class differences corresponds to a system of lifestyle differences, and that it is these class-determined lifestyle differences which underpin structural exclusion processes (Hjellbrekke, Jarness, & Korsnes, 2015, p. 197). This process takes place through ‘habitus’, an acquired system of dispositions formed in the context of people’s social locations (Williams, 1995, p. 585). Bourdieu explored how culture relates to social inequality and how the pursuit of distinction or differential recognition shapes all realms of social practice (Bourdieu, 1984). Although he did not write directly on health, Bourdieu showed how health and lifestyles are caught up in struggles for social recognition (Williams, 1995, p. 599). Whilst some critics have suggested his model is too deterministic, Bourdieu argues that habitus is an open system in which experiences constantly affect and modify dispositions (Bourdieu & Wacquant, 1992, p. 132). One instance of this flexibility is the idea of *‘*cleft habitus’, which Bourdieu uses to describe a mismatch whereby the individual experiences dissonance and does not feel ‘at home’ in their class habitus, typically because of social mobility (Bourdieu, 2007, p. 100; Friedman, 2016); I will return to this idea in relation to e-cigarette use. Although I have referred to classed practices, Bourdieu resisted the reification of rigid classes and saw class as essentially relational. Whilst Bourdieu refers to the dominant and the dominated classes, for the purposes of this paper I will use ‘working-class’ as a broad term to indicate people engaged in manual and routine jobs, and ‘middle-class’ as a contrasting term.

### Bourdieu’s distinction and the social gradient in smoking

Bourdieu points out that cultural practices can change their meaning over time, for instance by becoming associated with lower or higher class (Bourdieu, 1998; Hjellbrekke et al., 2015, p. 190). In this first section, I analyse just such a historical evolution of taste, namely the social gradient in smoking. Although concerns about the effect of tobacco on health have been expressed since the early stages of its diffusion into Western Europe (James, 1954 [1604]), it was the introduction of bright leaf, flue-cured inhalable tobacco in 1839 and the cigarette machine in 1881 (Brandt, 2009, p. 24–27) which led to the public health disaster of the 1950s and 1960s when the consequences of greater ease of smoking and deeper inhalation became apparent in increased rates of lung cancer, previously a rare disease (Doll & Hill, 1950). Since that time smoking has primarily been studied as a public health problem involving the mapping of continued smoking patterns and the design and evaluation of interventions designed to decrease smoking prevalence.

Tobacco use in high-income countries is characterised by a social gradient whereby socio-economic status is inversely correlated with smoking (Barbeau, Krieger, & Soobader, 2004; Blackwell, Lucas, & Clarke, 2014; Hiscock, Bauld, Amos, & Platt, 2012; Reid, Hammond, & Driezen, 2010); Lopez’s tobacco epidemic model (Lopez, Collishaw, & Piha, 1994) suggests that cigarette smoking first spread among the most powerful groups, starting with middle-class men then becoming more common across all classes and amongst women. Once smoking became widespread, middle-class men then middle-class women ceased smoking, whilst the least powerful continued to smoke (Dixon & Banwell, 2009, p. 2207). The point of the model is to help predict stages of the tobacco epidemic in countries thought to be in its earlier stages, and try to put measures in place to short-circuit its further development (Cairney, Studlar, & Mamudu, 2011, p. 232).

Whilst the Lopez model still has predictive power (Thun, Peto, Boreham, & Lopez, 2012), it does not explain the mechanisms behind the temporal trends it describes. Social scientists, most notably Pampel, have suggested that cigarettes were taken up initially by the middle-class to differentiate themselves from the working-class, then abandoned by them for the same reason (Ferrence, 1989, Ferrence, 1996, Pampel, 2005, Pampel, 2010). Pampel’s analysis of US data concluded that ‘smoking declines first among high status persons, who become concerned with health, fitness, and the harm of smoking, and separate themselves from other groups by rejecting smoking and other unhealthy status’ (Pampel, 2005, p. 120). Paralleling Pampel’s quantitative analysis is Poland’s qualitative work with smokers and non-smokers; he found that ‘the dominant classes recast as distinctive and worthy of emulation their own rejection of (cigarette) smoking, their smoke-free status’ (Poland, 2000, p. 10). These Bourdieuian analyses argue that being smoke-free confers distinction; smoking is rejected by the middle-class not only or primarily because it is objectively unhealthy, but because it has become associated with working-class status.

Despite the explosion of interest in Bourdieu in the social sciences (Outhwaite, 2009), Pampel and Poland’s characterisation of the rejection of smoking as an example of class distinction has achieved limited currency. It is generally argued that the decline in smoking resulted from the dissemination of medical findings and the development of tobacco control (Berridge, 2007, Berridge, 2013, Brandt, 2009) through coalitions of influence which affected public opinion and policy (Cairney et al., 2011; Feldman & Bayer, 2004; Rabin & Sugarman, 2001). The two explanations are not mutually exclusive, since social norms are themselves influenced by policy (Marmor & Lieberman, 2004, p. 275), and as Berridge argues in relation to the post-war decline in smoking in the UK, ‘the thresholds for public regulation and intervention were themselves social and political and both reflected and reacted upon culture’ (Berridge, 2013, p. 187); she also points out that health education can be effective only if it builds on ‘issues already inherent in culture’ (Berridge, 2013, p. 152).

### Smoking and class stigma

As the middle-class moved away from smoking to distinguish themselves from the working-class, a circular process took place whereby smoking became ever more stigmatised in middle-class circles, leading to ever more middle-class cessation. Stigma involves the rejection of particular people because of attributes which are not acceptable to their wider society; the process results in ‘spoiled identity’ (Goffman, 1963) and depends on the existence of a power differential which allows labelling, stereotyping, separation, status loss and discrimination to take place (Link & Phelan, 2001, p. 382). In the UK, the stigma attached to poverty (Jones, 2011, Lawler, 2005) meant that as elites abandoned smoking which became a habit only of the poor, class stigma and smoking stigma became mutually reinforcing. Public health campaigns used the ‘pedagogy of disgust’ (Lupton, 2015) to reinforce a class-based notion of smokers (Frohlich, Mykhalovskiy, Poland, Haines-Saah, & Johnson, 2012, p. 981). In the US, whilst poverty is stigmatised as a failure to achieve the American dream (Lamont, 2009, Sennett and Cobb, 1972), the association between poverty and smoking has been less clear than in the UK because of cross-cutting patterns of smoking by race, gender and acculturation (Barbeau et al., 2004; Kawachi, Daniels, & Robinson, 2005; Navarro, 1990). However, morality plays a key role in American public life and health policy (Morone, 1997, Morone, 2004), and although it has taken longer in the US for smoking to be explicitly linked with poverty (Wan, 2017), it has long been constructed as immoral and disgusting (Rozin, 1999; Rozin & Singh, 1999). Similar processes conflating poverty and smoking stigma have taken place in other high-income countries (Peretti-Watel, Legleye, Guignard, & Beck, 2014; Thompson, Barnett, & Pearce, 2009; Triandafilidis, Ussher, Perz, & Huppatz, 2016).

The operation of distinction also explains why tobacco control in high-income countries has accelerated, becoming more active and successful in the past fifteen years (Berridge, 2007, Smith, 2013b); many middle-class policy-makers still smoked in the initial period, whereas only the poor smoked later on: the gradual conflation of class and smoking stigma made stronger action against tobacco possible. Brandt suggests there may be a ‘tipping point’ for stronger tobacco control based on the changing ratio of smokers to non-smokers (Brandt, 2004, p. 34); Berridge points out that once a substance is connected with a non-mainstream group, further discussion embodies a distancing and fear of ‘the other’ (Berridge, 2013, p. 78). However, whilst there has been considerable literature on the ethics of using stigma as a public health tool (Bayer & Stuber, 2006; Burris, 2008; Chapman & Freeman, 2008; Stuber, Galea, & Link, 2008; Stuber, Galea, & Link, 2009; Williamson, Thom, Stimson, & Uhl, 2014), few studies have made the point that smoking stigma operates as a place-holder or proxy for class stigma, which it exploits and exacerbates (exceptions are Farrimond & Joffe, 2006, p. 487; Graham, 2012, p. 92–93).

Against this argument, it might be suggested that studies of the experience of social disapproval by smokers have shown no consistent pattern by class status (Ritchie, Amos, & Martin, 2010). Stuber et al. (2008) found less experience of smoking stigma among lower-status compared to higher-status smokers (Stuber et al., 2008), whereas Farrimond and Joffe (2006) found more experience of stigma amongst lower-status smokers – particularly in contexts where non-smoking was the norm – and also that higher-status respondents were more likely to conceal their smoking (Farrimond & Joffe, 2006, p. 486–487). I suggest the explanation lies in the fact that the smoking gradient is spatialized, so that people of contrasting class and smoking status live in culturally and geographically separate social and spatial communities (Barnett, Moon, Pearce, Thompson, & Twigg, 2017, p. 34; Fahmy, Gordon, Dorling, Rigby, & Wheeler, 2011; Poland, 2000, p. 11; Wacquant, 2007). Individual experiences of stigma therefore depend on the extent to which the stigmatised behaviour is performed outside safe spaces of acceptance (Glenn, Lapalme, McCready, & Frohlich, 2017). Poor residents of ‘smoking islands’ (Thompson, Pearce, & Barnett, 2007) are segregated from middle class enclaves, so lower-status smokers encounter little stigma in their own, high-smoking neighbourhoods. The small number of higher-status smokers conceal their smoking or differentiate their own occasional ‘social’ smoking from the stigmatised daily smoking of class others (Choi, Choi, & Rifon, 2010; Hoek, Maubach, Stevenson, Gendall, & Edwards, 2013; Nichter, 2015; Sæbø, 2016; Schane, Glantz, & Ling, 2009).

### Children and bystanders

The residualization of smoking as stigmatised, classed behaviour led to a new emphasis on the rights and health of children and bystanders rather than smokers, who became ‘the other’ (Graham, 2012, p. 87 & 92). Whilst the discovery of the harmful effects of second-hand smoke (Hirayama, 1981) was a key factor in the implementation of smoking bans in public places (Hyland, Barnoya, & Corral, 2012), the operation of stigma was also crucial. The health hazards of environmental tobacco smoke were a ‘scientific fact waiting to emerge’ (Berridge, 1999) since health pressure groups were already arguing it before there was any scientific evidence for it causing harm (Gostin, 1997, p. 346; Smith, 2013a, p. 63–68). Berridge described the authority of science ‘changing a moral issue into a scientific one, albeit with continuing moral overtones’ (Berridge, 2004, p. 125); the UK smoking ban had symbolic significance in marking a continuing detachment of tobacco from mainstream culture (Berridge, 2013, p. 237).

The idea of smoking as not so much a matter of individual choice as a threat to *‘*innocent victims’ (Berridge, 2004, p. 25) was particularly useful in the US as a way for tobacco control advocates to get round American fears of government interference with individual freedoms (Bayer & Colgrove, 2004, p. 34). The US has a tradition of appealing explicitly to moral considerations in public policy (Cairney et al., 2011, p. 131) and the new focus meant smokers could be construed as guilty not just of an individual failure of self-control, but of wilful endangerment of others. In both the US and UK, smoking became medicalised as addiction (Bayer & Colgrove, 2004, p. 36), but policy responses diverged. For the US, addiction was a moral failing with abstinence as the correct solution, whereas the UK saw addiction as removing agency – and therefore blame – from the smoker and took a harm reduction approach, including the prescription of nicotine replacement therapy (Berridge, 2007, p. 241–278; Green, Bayer, & Fairchild, 2016). Green et al. (2016) suggest that it is this difference in focus – reducing harm to smokers in the UK, versus protecting children and bystanders in the US – which determines UK and US currently prevailing attitudes to e-cigarettes (Green et al., 2016, p. 1303). Framing e-cigarettes in different ways constructs them either as reducing smoking prevalence and addressing health inequalities by helping smokers quit, or as posing a risk to non-smoking children and young people; supporters of e-cigarettes focus on existing smokers whereas opponents are concerned about e-cigarette uptake by young people who would not otherwise smoke (McKee & Capewell, 2015, p. 1).

An additional reason why tobacco control policy tends to focus on young people is the fact that the social gradient in smoking is generally less pronounced among younger people than in later adulthood, fuelling concern about young smokers as a stand-alone category. I argue however that superficially similar smoking rates amongst working- and middle-class young people conceal radically different motivations: middle-class young people smoke as a temporary rebellion against middle-class values (Ehrenreich, 1990; Ortner, 2006) whereas working-class young people smoke to claim adulthood rather than to challenge parental or collective values (see also Holdsworth, 2009 for contrasting classed meanings of youth transitions). Ortner follows Bourdieu in arguing that classes are relational, defining themselves always in implicit reference to the other (Ortner, 2006, p. 27). Middle-class parent-child relations, she suggests, are riven by the fear of downward mobility: parents attempt to control children, whose possible failure embodies the threat of a working-class future (Ortner, 2006, p. 31), whilst children resist their parents’ values through symbols of lower-class affiliation, which I suggest include smoking.

### Smokers and e-cigarettes

Having argued for a Bourdieuian reading of the evolution of policy on smoking and cessation, I now apply his thinking to smoking, cessation and e-cigarette use in the field of classed cultural practices. I start with the small number of middle-class, residualised smokers who are typically conflicted about their smoking; as we saw earlier, they find ways of deflecting stigma through secret smoking or by defining social smoking as a separate practice. I account for their continued existence with reference to what Bourdieu called the ‘cleft’ habitus, a cognitive dissonance which prevents them feeling completely at home in their class position, perhaps because of recent class mobility; I suggest that one way this dissonance is expressed is through an inability or unwillingness to quit smoking, since a cleft habitus in terms of class position potentially corresponds to a similarly cleft habitus in terms of cultural practices. The advantage of the e-cigarette is that it provides a way of resolving this dissonance by supplying similar cultural meanings without the obvious health risks of tobacco. Upwardly mobile smokers fit this category of cleft habitus (Friedman, 2016), as do those middle-class smokers engaged in work or leisure pursuits which have their roots in working-class culture (e.g. various musical subcultures). This demographic arguably represents the most vocal and visible e-cigarette use and hence has shaped the image of e-cigarettes (Smith, 2015). Smoking cessation as distinction tends not to operate for smokers whose identification with mainstream middle-class values is weak, but e-cigarettes provide them with an alternative symbol of outsider status without compromising the middle-class imperative of health (Lupton, 1995). As smokers, this demographic suffered cognitive dissonance and could not ‘talk back’ to stigma (Hooks, 1986), but as e-cigarette users, they regain the moral high ground and their anger regarding regulatory threats is no longer constrained by shame.

I now turn to working-class smokers and the prospects for reducing health inequalities through the large-scale substitution of-cigarettes for tobacco. Smoking fits into the working-class habitus of sociable hedonism which Bourdieu described, notably in relation to eating practices (Bourdieu, 1984, p. 180, 183, 394). Sociable hedonism, he argues, goes hand in hand with the rejection of middle class practices seen as ‘pretentious’ including excessive attention to one’s health or appearance. Drawing on her own fieldwork, Skeggs refers in similar terms to the creative hedonism and anti-pretentious humour of working-class culture (Skeggs, 2004, p. 88; Skeggs, 2011, p. 506). Those who transgress are ‘called to order’ (Bourdieu, 1984, p. 380) through mockery, and reminded of the need for class solidarity (Bourdieu, 1984, p. 381). Men in particular, Bourdieu notes, ‘are forbidden every sort of “pretension” in matters of culture, language or clothing’ (Bourdieu, 1984, p. 382). I suggest that smoking cessation is potentially pretentious in this sense of excessive attention to the self; would-be quitters are tempted or bullied back into smoking by their friends (Thirlway, 2015). In Bourdieu’s words, ‘Not the slightest deviation is permitted to those who belong to the same class (or originate from it), because in this case difference could only arise from the desire to distinguish oneself, that is, from refusal or repudiation of the group’ (Bourdieu, 1984, p. 381).

Although smoking cessation is difficult to reconcile with sociable hedonism, there are other aspects of the working-class habitus which are more conducive to quitting, notably those which Bourdieu characterised as invoking solidarity and community (Bourdieu, 1984, p. 183; Skeggs, 2004). Thus, although excessive attention to personal health is pretentious, a competing ethos of family responsibility makes it a moral duty to stay healthy to meet one’s family obligations. A clear health threat correlates with smoking cessation (Gallus et al., 2013), and the smoker who does not quit after contracting a serious illness suffers blame (Chapple, Ziebland, & McPherson, 2004; Gullick & Stainton, 2006). This familial aspect of the working-class habitus typically comes into play at key transitions such as becoming a parent or developing a health problem (Nichter et al., 2007; O’Brien, Hunt, & Hart, 2009; Thirlway, 2015). Sociable hedonism may still take over at times, for instance when the would-be quitter is out drinking with friends (Lucherini, Rooke, & Amos, 2017, p. 4–5), and the family narrative may not be available to those who are socially isolated (Giordano & Lindström, 2011), which is consistent with low rates of smoking cessation amongst groups lacking social ties, such as unemployed and homeless people (Borrelli, 2010).

Turning now to the role of e-cigarettes in working-class smoking cessation, whilst their use can tap into the ethos of smoking cessation as family responsibility which I have described, in this narrative the e-cigarette does not represent youthful fun, but maturity and the assumption of adult responsibilities; this type of e-cigarette user tends to avoid sophisticated devices and exotic flavours. Where e-cigarettes differ from other cessation aids, however – or indeed, stop being cessation aids at all – is in also being compatible with the working-class ethic of sociable hedonism to which Bourdieu referred. This results from their ‘gadget’ allure, their association with pleasure through the many attractive and colourful models and flavours available, and the potential for play in doing tricks or creating vapour clouds. This translates into a division between two types of working-class-cigarette user, whom I characterise as the ‘practical family quitter’ focused on cessation and largely uninterested in or even hostile to e-cigarettes as recreation or lifestyle, and the ‘recreational user’ primarily interested in pleasure and play, typically younger and male (Farrimond, 2017, Thirlway, 2016). Whilst individuals in this second group may substitute e-cigarettes for tobacco, they may also choose to continue using both.

## Conclusions

In the first part of this article, I built on Bourdieu’s theory alongside scholarship from a range of disciplines to argue that the historical evolution of policy and practice on smoking and cessation in high-income countries has been shaped by class. I described how smoking stigma came to be a proxy for class stigma and suggested that this explains why middle-class smokers are more likely than working-class smokers to quit successfully (Kotz & West, 2009). Most research makes the middle-class assumption that smoking cessation is the only rational choice, and therefore focuses on why working-class smokers find it harder to quit rather than why middle-class smokers find it easier (Hiscock, Judge, & Bauld, 2011), but class disgust is a powerful emotion (Farrimond & Joffe, 2006; Lawler, 2005; Rozin, 1999; Rozin & Singh, 1999) and one which I suggest is key to middle-class smoking cessation. Lupton argues that using disgust and the exclusion of particular social groups to change health behaviour is unethical (Lupton, 2013, Lupton, 2015); but it is also ineffective for working-class smokers, since the deployment of class disgust can, by definition, only resonate with the middle-class.

As well as the stigmatisation of smokers as ‘class other’, I attributed the preoccupation with young people’s substance use which is a key feature of opposition to e-cigarettes to a long-standing moral panic about the symbolic adoption by middle-class young people of working-class cultural practices. Smoking as rebellion against the pressure for educational success inherent in the middle-class’s need to reproduce its own position has resulted in youth smoking being less stratified by class than adult smoking, creating the idea of the ‘youth smoker’. This analysis led me to question the usefulness of ‘youth’ as a category of analysis and therefore also the relevance of concerns about young people’s e-cigarette or tobacco use outside a class framework. Historicizing Ortner and Ehrenreich’s argument, I tentatively suggest that lower rates of youth smoking in recent years may map to greater anxiety about their future amongst middle-class young people, and hence a lessened propensity to contest parental values of class reproduction than in the period of near-full employment in which Ortner and Ehrenreich grew up.

In a second part, I applied Bourdieu’s thinking to smoking, cessation and e-cigarette use as classed cultural practices, arguing that residual middle-class smokers in high-income countries are often those who experience a ‘cleft habitus’ or anomalous class position and hence exhibit anomalous class practices including smoking; e-cigarette use can resolve this contradiction as a practice sufficiently similar to smoking but without the obvious health risks. For working-class smokers, e-cigarettes are compatible with an ethos of smoking cessation as family responsibility and may function as a smoking cessation aid in this context; they are also compatible with an ethos of sociable hedonism, but as a pleasure and not necessarily as a smoking cessation device. My classification of e-cigarette users into contrasting types is consistent with the few studies which include working-class voices (Farrimond, 2017; Lucherini et al., 2017; Rooke, Cunningham-Burley, & Amos, 2016; Thirlway, 2016) and I suggest that qualitative research has a unique role to play in illuminating e-cigarette user motivations across the class spectrum. The first category I identified, the middle-class (or socially mobile) ‘moral high ground’ e-cigarette user, is already highly visible, but little has been written about the two working-class categories i.e. the ‘practical family quitter’ focused on e-cigarette use as smoking cessation and the ‘recreational user’, often placed in the morally ambiguous category of ‘dual user’, with motivations which remain obscure and highly contested (Maglia, Caponnetto, Di Piazza, La Torre, & Polosa, 2017).

As regards the potential of e-cigarettes to facilitate smoking cessation in the working-class and thereby reduce health inequalities, I have argued that the way that the struggle for distinction has played out in relation to smoking has resulted in middle-class smokers having – in class disgust – a stronger reason to quit than working-class smokers. Nevertheless, I suggest there is potential for tobacco control to tap into the ethos of family care and responsibility which I have described, and both smoking cessation and e-cigarette use may resonate with other aspects of working-class habitus which I have not discussed here.

Turning to Bourdieu scholarship in more general terms, I suggest my analysis develops the Bourdieuian concept of habitus in three ways. The first is cleft habitus, a concept which has generally been used to describe the ambiguous experience of social mobility (Friedman, 2016; Thatcher, Ingram, Burke, & Abrahams, 2015, chap. 8 and 10) but which I have demonstrated can also illuminate cultural practices. The second is my application of Ortner’s and Ehrenreich’s Bourdieuian accounts of how middle-class young people depart from class habitus as a form of generational rebellion, which I suggest has the potential to unsettle other instances of what are thought to be cross-class youth practices and ultimately to question the relevance of youth as a useful category of analysis independent of class. Finally, I placed new emphasis on the active avoidance of middle-class pretension (Skeggs, 2014) and the importance of family responsibility, relationality and care as neglected aspects of working-class habitus (Reay, 2004, Reay, 2015; Skeggs, 2004, Skeggs, 2011; see also Warin, Turner, Moore, & Davies, 2008 on working-class mothers’ relational identities and obesity).

## Conflict of interest

No financial disclosures to report.
